# Atypical Complications during the Course of COVID-19: A Comprehensive Review

**DOI:** 10.3390/medicina60010164

**Published:** 2024-01-15

**Authors:** Tauqeer Hussain Mallhi, Aqsa Safdar, Muhammad Hammad Butt, Muhammad Salman, Sumbal Nosheen, Zia Ul Mustafa, Faiz Ullah Khan, Yusra Habib Khan

**Affiliations:** 1 Department of Clinical Pharmacy, College of Pharmacy, Jouf University, Sakaka 72388, Saudi Arabia; yhkhan@ju.edu.sa; 2 Faculty of Pharmaceutical Sciences, University of Central Punjab, Lahore 54000, Pakistan; l1f20mpch0005@ucp.edu.pk; 3 Department of Medicinal Chemistry, Faculty of Pharmacy, Uppsala University, 75123 Uppsala, Sweden; 4 Institute of Pharmacy, Faculty of Pharmaceutical and Allied Health Sciences, Lahore College for Women University, Lahore 54000, Pakistan; msk5012@gmail.com; 5 Department of Pharmacy, The Children’s Hospital and the University of Child Health Sciences, Lahore 54600, Pakistan; dr.sumbal@chich.edu.pk; 6 Department of Pharmacy Services, District Headquarter (DHQ) Hospital, Pakpattan 57400, Pakistan; zia.ucp@gmail.com; 7 Department of Pharmacy Administration and Clinical Pharmacy, School of Pharmacy, Xi’an Jiaotong University, Xi’an 710061, China; fkhan@bs.qau.edu.pk

**Keywords:** COVID-19, complications, gastro-intestinal system, neurological system, renal system, cardiovascular system

## Abstract

COVID-19 is primarily a respiratory disease, but numerous studies have indicated the involvement of various organ systems during the course of illness. We conducted a comprehensive review of atypical complications of COVID-19 with their incidence range (IR) and their impact on hospitalization and mortality rates. We identified 97 studies, including 55 research articles and 42 case studies. We reviewed four major body organ systems for various types of atypical complications: (i) Gastro-intestinal (GI) and hepatobiliary system, e.g., bowel ischemia/infarction (IR: 1.49–83.87%), GI bleeding/hemorrhage (IR: 0.47–10.6%), hepatic ischemia (IR: 1.0–7.4%); (ii) Neurological system, e.g., acute ischemic stroke/cerebral venous sinus thrombosis/cerebral hemorrhage (IR: 0.5–90.9%), anosmia (IR: 4.9–79.6%), dysgeusia (IR: 2.8–83.38%), encephalopathy/encephalitis with or without fever and hypoxia (IR: 0.19–35.2%); (iii) Renal system, e.g., acute kidney injury (AKI)/acute renal failure (IR: 0.5–68.8%); (iv) Cardiovascular system, e.g., acute cardiac injury/non-coronary myocardial injury (IR: 7.2–55.56%), arrhythmia/ventricular tachycardia/ventricular fibrillation (IR: 5.9–16.7%), and coagulopathy/venous thromboembolism (IR: 19–34.4%). This review encourages and informs healthcare practitioners to keenly monitor COVID-19 survivors for these atypical complications in all major organ systems and not only treat the respiratory symptoms of patients. Post-COVID effects should be monitored, and follow-up of patients should be performed on a regular basis to check for long-term complications.

## 1. Introduction

Coronavirus disease 2019, or COVID-19, is an exceedingly transmissible viral illness that is acquired by severe acute respiratory syndrome coronavirus 2 (SARS-CoV-2). It has had a disastrous consequence on the world, culminating in over six million deaths around the globe. Succeeding the early cases of this dominating respiratory viral disease, which were reported in December 2019 in Wuhan, Hubei Province, China, SARS-CoV-2 swiftly promulgated around the globe in a brief period of time. As a result, the World Health Organization (WHO) was forced to announce it as a global pandemic on 11 March 2020, and trials for the first human vaccine for COVID-19 started with the modern mRNA vaccine. By April 2020, 1 million cases were reported, and the WHO released guidance for mask-wearing. The major countries affected included the USA, the UK, India, Russia, and Vietnam. By September 2020, 1 million deaths were recorded. In November 2020, vaccine trials of Pfizer and BioNTech showed 90% efficacy. By April 2021, one billion doses of COVID-19 vaccination were given all across the world. According to recent data, three million new cases and more than 23,000 deaths have been reported from 13 March to 9 April 2023, a decline of 28% and 30%, respectively, in comparison to the preceding 28 days. As of 9 April 2023, over 762 million confirmed cases and over 6.8 million deaths have been reported all over the world [1,2,3].

According to its structure and phylogenetics, SARS-CoV-2 is identical to MERS-CoV and SARS-CoV. It is made up of four major structural proteins: membrane protein (M), nucleocapsid (N), envelope (E), and spike (S), together with sixteen nonstructural proteins and five to eight accessory proteins [4]. The surface spike glycoprotein (S) consists of an S1 subunit, which is divided further into the receptor-binding domain (RBD) and the N-terminal domain (NTD), which aids the virus to enter into the host cell and acts as a prospective target for neutralization concerning vaccines or antisera [5]. SARS-CoV-2 gains access into the host cell once its spike (S protein) abundantly binds to angiotensin-converting enzyme 2 (ACE2) receptors present on the respiratory epithelium, for example, type Ⅱ alveolar epithelial cells. The amino acid site of the spike RBD permits functional processing of a similar kind in the presence of the human enzyme furin, which allows the amalgamation of viral and cell membranes, a vital transit for the virus’s entry into the cell. This is followed by the subsequent endocytosis and viral replication along with virion assembly [6]. In addition to the respiratory epithelium, ACE2 receptors are also present in other organs such as enterocytes and the proximal tubular cells present inside the kidney, ileum, upper esophagus, myocardial cells, and the urothelial cells that make up the bladder [7].

Primarily, COVID-19 is considered a viral respiratory as well as vascular disease, as SARS-CoV-2 principally targets the respiratory and vascular systems. In spite of the fact that the respiratory system is the main target of SARS-CoV-2, it causes atypical complications in systems such as the renal, cardiovascular, hepatobiliary, gastrointestinal tract (GI), and central nervous systems [8]. SARS-CoV-2-prompted organ dysfunction, by and large, is possibly described by one or a combination of the suggested mechanisms, such as dysregulation of the renin–angiotensin–aldosterone system (RAAS), direct viral toxicity, dysregulation of the immune system, ischemic injury resulting from thrombo-inflammation, thrombosis, and vasculitis [9].

Considering the large volume of research data on the atypical complications of COVID-19, it is crucial to perform an overview so that current literature can be organized and identified to underline the scope of priority for successful clinical management and effective decision making by healthcare practitioners to not only curb the impact of these atypical complications on the patient’s quality of life but also to reduce the economic burden on the healthcare system. Previously, there have been a few reviews on extrapulmonary manifestations [8,10,11,12,13], but the types of complications and their overall effect on hospitalization and mortality rates have never been explored. In addition, a large number of reviews exist on manifestations in individual organ systems of the body [14,15,16,17,18,19], but no study has ever attempted to gather information in a single article through extensive research on major organ systems. Furthermore, there are other infections that can cause atypical complications, i.e., dengue and Varicella-Zoster virus infections are well known to cause dementia, chronic encephalitis, aseptic meningitis, multiple sclerosis, acute pancreatitis, and myopericarditis [20,21,22,23,24,25] and such complications further accelerate patient hospitalization and rates of morbidity and mortality, which, in turn, render a huge burden on the healthcare system.

## 2. Purpose of Article

This review was intended (1) to summarize a large number of research studies and case reports on various atypical complications of the COVID-19 virus in major organ systems, (2) to assemble the data on the range of their incidence, and (3) to gather information on the impact of these complications on hospitalization and mortality rates.

## 3. Review Method

This article is not subject to ethical approval as it includes the synthesis of qualitative data from publicly available information. Inclusion criteria included articles that were either research studies or case studies related to atypical complications during or after SARS-CoV-2 diagnosis in four major organ systems, i.e., the gastrointestinal and hepatobiliary system, renal system, neurological system, and cardiovascular system. Research studies reporting the types of complications in the aforementioned organ systems with either incidence rates, mortality/hospitalization rates, or both were included in this review. Articles reporting atypical complications in diagnosis other than SARS-CoV-2 infection (e.g., HIV, Dengue, Varicella-Zoster virus) were excluded as their mechanisms for organ complications synergize with those of the SARS-CoV-2 virus. Articles that were not concerned with the preceding organ systems or had repetitive complications were also excluded.

PubMed, Scopus, EMBASE Cochrane Library, Medline, and ProQuest databases were searched for studies published between January 2020 and May 2023. A search strategy utilizing an extensive range of Medical Subject Headings (MeSH) was employed: “Atypical and extrapulmonary manifestations” [Mesh] OR “Atypical and extrapulmonary features” [Mesh] OR “Atypical symptoms” [Mesh] OR “Atypical complications” [Mesh] OR “Extrapulmonary complications” [Mesh] AND (“COVID-19” [Majr] OR “SARS-CoV-2” [Majr] OR “Coronavirus” [Majr] OR “Coronavirus pandemic” [Majr]) AND (“Atypical” [Mesh] OR “Multiple organ manifestations” [Mesh] OR “Extrapulmonary features” [Mesh]).

All records were checked by independent reviewers on the basis of titles and abstracts for the purpose of addition to this review. Studies that did not harmonize the inclusion criteria or those that harmonized the exclusion criteria were discarded. The remaining records with abstracts providing adequate information were taken into consideration after the evaluation of the full text, which was carried out by the same reviewers independently. The reference lists of full-text articles were also screened for additional studies. Disagreements were solved by a third reviewer.

In this review, only studies were included that provided variable results so that the range of incidence of the identified complications could be recorded. For example, if a study reported the incidence of acute kidney injury as 0.5% and another study reported 0.57%, in order to determine the lowest incidence, only one study was considered so as to avoid the repetition of studies providing similar information. A similar method was adopted for the highest limit of the incidence rate. Also, where a particular complication was reported in 2 or 3 studies only, the incidence is reported separately instead of the incidence range (IR). Since this review aims to cover many complications, it was not possible to cover the enormous literature available to date.

The studies that were selected were scrutinized by two investigators, and the information was extracted by using the standardized system. The information that was gathered from research studies is as follows: author name, year of publication, country, type of study, demographics, complications, number of complications, incidence rate, and hospitalization and mortality rate. The following data were extracted from case reports: author name, publication year, demographics, type of complication, past history, laboratory tests (latest test values were noted where available), treatment, days of illness before hospitalization, hospital stay, and outcome.

## 4. Summary of Included Studies

Of 65,179 total studies, 17,880 studies were initially selected. Of these, 4365 studies were excluded after abstract screening. A total of 1513 full-text articles were assessed for eligibility. Finally, 55 research studies and 42 case reports were included in this review. The current review has identified complications pertaining to four major organ systems: (1) Gastrointestinal/hepatobiliary system, (2) neurological system, (3) renal system, and (4) cardiovascular system. Details of complications identified from the selected studies along with underlying pathophysiology and management considerations are described below.

### 4.1. Gastrointestinal System

#### 4.1.1. Complications

**Figure 1:**
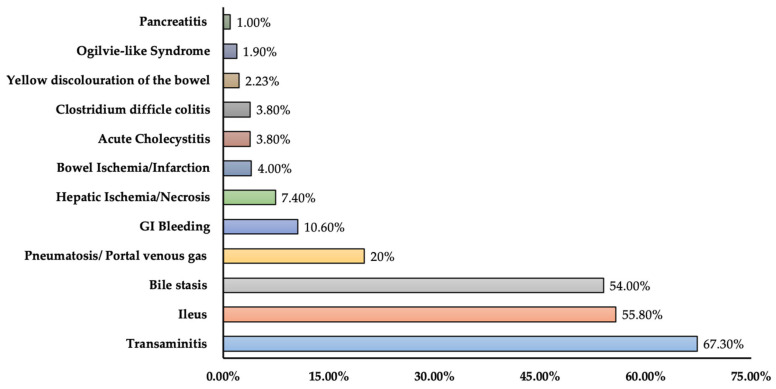
Prevalence of gastrointestinal complications among COVID-19 patients (prevalence represents the maximum percentage reported in the literature).

In this review, seven research studies have been identified in which GI complications were reported. There was a total of 14 GI complications identified from research studies. The major complications were bowel ischemia/infarction (IR: 1.49–4.00%), GI bleeding/hemorrhage (IR: 0.47–10.6%), and hepatic ischemia/injury/infarct due to thromboembolism of the portal system (IR: 1.0–7.4%). Other complications included acute pancreatitis (incidence: 0.3%, 1.0%), transaminitis (incidence: 55%, 67.3%), and ileus (incidence: 48.0%, 55.8%). The rare complications identified were luminal and pancreaticobiliary disease (incidence: 2.7%), pneumatosis or portal venous gas (incidence: 20%), yellow discoloration of the bowel (2.23%), bowel-wall abnormalities (31%), bile stasis (incidence: 54.0%), acute cholecystitis (incidence: 3.8%), gastric feeding intolerance (incidence: 46.2%), Ogilvie-like Syndrome (incidence: 1.9%), and *Clostridium difficile* colitis (incidence: 3.8%). The overall mortality rate among research studies reporting GI complications ranged from 25% to 34.1% (Table 1). In Figure 1, the prevalence in terms of the maximum percentage of GI complications among different studies is presented.

We included 14 case reports comprising 19 patients identified with GI complications. Of these, major complications were bowel ischemia/hepatic ischemia (38.88%), acute pancreatitis (22.22%), and bowel perforation (16.67%). Rare complications were Cytomegalovirus hemorrhagic enterocolitis (11.12%), paralytic ileus (11.12%), and intussusception (5.56%). Of case reports identifying GI complications, nine (50.0%) patients died, five (27.77%) patients were cured, and four (22.22%) patients were still hospitalized and undergoing treatment (Table 2).

#### 4.1.2. Underlying Pathophysiology

The underlying pathophysiology related to GI damage among COVID-19 patients is most likely multifactorial. The most credible mechanism is direct virus-mediated tissue damage due to the existence of ACE2 receptors in the glandular cells of the intestine [26,27,28,29], in addition to the assimilation of the viral nucleocapsid protein among the epithelial cells of the stomach, duodenum, and rectum, as well as glandular enterocytes [27]. Additionally, diffused endothelial inflammation among the submucosal vessels of the small intestine in histopathological evidence and mesenteric ischemia suggest microvascular small-bowel injury [30]. The existence of infiltrating lymphocytes and plasma cells and interstitial edema in the gastric, duodenal, and rectal lamina propria of COVID-19 patients provides support for tissue damage mediated by inflammation [27]. It has also been suggested that the GI manifestations and severe disease progression are contributed by the modification of the intestinal flora caused by the virus [31].

#### 4.1.3. Management Considerations

Specific management considerations should be considered by health practitioners, which should include differential diagnosis for COVID-19 among patients suffering from isolated GI symptoms in the non-appearance of respiratory symptoms [32]. Practitioners should prioritize testing for COVID-19 among patients presenting both GI and respiratory symptoms if testing resources are limited [33], and diagnostic endoscopy should only be utilized for emergency therapeutic reasons such as biliary obstruction or large-volume GI bleeding [34,35]. Hepatic transaminases should be monitored longitudinally, in particular among patients who are given investigational treatments. Decreased levels should not particularly be considered a contraindication when treating with tocilizumab, lopinavir, and remdesivir [36].

**Table 1:** Summary of research studies reporting gastrointestinal complications in COVID-19 patients.

Author Name, Year	Country	Type	Demographics	Complications Reported	Incidence	Mortality
El Moheb et al., 2020 [37]	United States	Retrospective cross-sectional	N = 92, Male: 52 (57%) Age: COVID-19 ARDS vs. non-COVID-19 ARDS: 64.5 (51.5–75.5) vs. 62 (48.5–71.5) (*p* = 0.24)	Transaminitis	COVID-19 ARDS vs. non-COVID-19 ARDS: 55% vs. 27% (*p* < 0.001)	COVID-19 ARDS vs. non-COVID-19 ARDS: 25% vs. 23% (*p* = 0.73)
				Severe ileus	48% vs. 22% (*p* < 0.001)	
				Bowel ischemia	4% vs. 0% (*p* = 0.04)	
Mauro et al., 2021 [38]	Italy	Retrospective cross-sectional	N = 23, Male: 18 (78.26%) Age: 75 years (IQR: 64–78).	Upper gastrointestinal bleeding (peptic ulcer)	0.47%	21.7%
Drake et al., 2021 [39]	United Kingdom	Prospective cohort	N = 41,025, Male: 21 758 (59.8%) Age: 71.1 years (SD: 18.7)	Gastrointestinal hemorrhage	1%	31.5%
				Pancreatitis	0.3%	
				Liver injury	7.4%	
Qayed et al., 2021 [40]	United States	Prospective cohort	N = 878 (Admitted to ICU), Male: 543 (61.8%) Age: 61.9 years (SD: 15.1)	Luminal and pancreaticobiliary	2.7%	34.1%
				Severe gastrointestinal bleeding	1.1%	
Bhayana et al., 2020 [41]	United States	Retrospective cross-sectional	N = 412 (224 abdominal imaging studies in 134 patients), Male: 241 (58%), Age: 57 years (Range 18–90)	Bowel-wall abnormalities	31% (Among CT images)	NA
				Pneumatosis or portal venous gas	Seen in 20% of CT images in ICU patients	
				Yellow discoloration of the bowel	2.23% of all abdominal imaging patients	
				Bowel infarction	1.49% of all abdominal imaging patients	
				Ischemic enteritis	1.49% of all abdominal imaging patients	
				Bile stasis	54% of ultrasound examinations in patients with liver laboratory findings	
Dane et al., 2020 [42]	United States	Retrospective cohort	N = 82 COVID-19 patients who underwent abdominopelvic ultrasound or CT), Male: 58 (71%), Mean age: 58.8 years (SD: 14.5)	Thromboembolic findings	11% (3 arterial, 1 venous, 4 infarcts, 1 portal vein thrombosis, and 2 lower extremity thrombosis)	NA
Kaafarani et al., 2020 [15]	United States	Retrospective cross-sectional	N= 141 (ICU patients), Male: 92 (65.2%), Median age: 57 (IQR: 47.70)	Transaminitis	67.3%	NA
				Acute cholecystitis	3.8%	
				Acute pancreatitis	1.0%	
				Hepatic ischemia and necrosis	1.0%	
				Gastric feeding intolerance	46.2%	
				Ileus	55.8%	
				Ogilvie-like syndrome	1.9%	
				Bowel ischemia	3.8%	
				GI bleeding	10.6%	
				*Clostridium difficile* colitis	3.8%	

Abbreviations: ARDS, acute respiratory distress syndrome; CT, computed tomography; NA, Not available.

**Table 2:** Summary of case studies reporting gastrointestinal complications in COVID-19 patients.

Name of Author, Year, Ref	Demographics	Type of Complication	Past History	Laboratory Tests	Treatment	Days of Illness before Hospitalization	Hospital Stays	Outcome
De Nardi et al., 2020 [43]	53-year-old male patient	Bowel perforation	Hypertension and paroxysmal supraventricular tachycardia	Hb: 12.5, %, LDH: 313, WBC: 12.100, lymphocyte count: 6.6, CRP: 104 mg/L	At start: Antiretrovirals, hydroxychloroquine, anakinra, broad-spectrum antimicrobial treatment levofloxacin. During treatment: Low-molecular-weight heparin, anticoagulant therapy, and broad-spectrum antibiotic, piperacillin–tazobactam, and fluconazole	9 days	23 days	Cured
Del Hoyo et al., 2020 [44]	61 years, female	Extensive splanchnic vein thrombosis, liver, mesenteric and splenic ischemia	No	CRP: 9.43 mg/L, platelet count (46,000/μL), D-dimer levels: 43,998 μg/mL. AST 2728 U/L, ALT 1065 U/L	Enoxaparin 1 mg/kg SC q12h	NA	3 days	Died
Karna et al., 2020 [45]	61 years, female	Superior mesenteric artery thrombosis, gut ischemia	Hypertension	Hb: 7.1 mg/dL, TPC: 311,000 microliters, TLC: 23,000 per cubic millimeter, NLR: 18.5, monocyte: 5, CRP: 282 per cubic millimeter, aPTT: 32.9, PT/INR: 19/1.7	High-flow nasal oxygen (HFNO), cefoperazone-sulbactam, prophylactic enoxaparin, pantoprazole, vitamin C, and zinc	NA	Approximately 12 days	Died after surgery due to septic shock
Carll, Rady et al., 2021 [46]	Middle-aged woman	Cytomegalovirus hemorrhagic enterocolitis	No	Plasma interleukin (IL)-6: 14.1, elevated troponin-T, lymphocytopenia, and markedly elevated d-dimer, CRP and LDH	Remdesivir, two units of SARS-CoV-2 convalescent plasma, and systemic hydrocortisone IL-6 antagonist tocilizumab Ganciclovir Foscarnet systemic corticosteroids Ustekinumab	10 days	209 days	Cured
De Barry et al., 2020 [47]	79-year-old female	Arterial and venous abdominal thrombosis, extended bowel ischemia	No	CRP: 125 mg/L, hyperleukocytosis: (12,600/mm^3^),	Laparotomy, thrombolysis and thrombectomy of the upper mesenteric artery	8 days	NA	Died
Chan et al., 2020 [48]	73-year-old male	Ischemic colitis	Hypertension and end-stage renal disease on hemodialysis	Hb: 5.6 g/dL, MCV: 86.1 fL, iron: 18 μg/dL, TIBC: 98 μg/dL, iron saturation: 18.4%, WBCs showed leucopenia 3.8 × 10^3^/μL, lymphopenia with an absolute lymphocyte count of 600/μL, lactic acid level: 2 mmol/L, d-dimer: 3239 ng/mL, ferritin: 1713.5: ng/mL, CRP: 14.2 mg/dL.	Bowel rest, intravenous fluids, antibiotics (ciprofloxacin and metronidazole), and anticoagulants, Ciprofloxacin and metronidazole, anticoagulant	3 days	5 days	Died due to cardiac arrest
Corrêa Neto et al., 2020 [49]	80-year-old female	Acute perforated abdomen	Systemic arterial hypertension and ischemic heart disease	Leukocytosis: 19,950 mm^3^, platelet count: 507,200 mm^3^, GOT: 23.00 U/L, GPT: 41.00 U/L, creatinine: 5.54 mg/dL, urea: 139 mg/dL. D-dimer: 1466.8 ng/dL, ferritin: 1199 ng/dL, creatine phosphokinase: 239.00 U/L	Tazocin 4.5 g 3×/day and Azithromycin 500 mg/day, Rectosigmoidectomy with terminal colostomy	10 days	NA	Died
Hadi, Werge et al., 2020 [50]	47-years old woman	Acute pancreatitis	No	Pancreas-specific plasma amylase: 173 U/L	Fluid resuscitation and intravenous antibiotics	72 days	Patient was still admitted	N/A
Hadi, Werge et al., 2020 [50]	68-year-old woman	Acute pancreatitis	Hypertension, hypothyroidism, and osteoporosis	Amylase level: 85 U/L	Fluid resuscitation and intravenous antibiotics	2 days	Patient was still admitted	N/A
Hadi, Werge et al., 2020 [50]	71-year-old man	Acute pancreatitis	No	NA	Invasive mechanical ventilation, intravenous antibiotics, noradrenaline, and dopamine	3 days	NA	Died
Marchi, Vianello et al., 2020 [51]	73-year-old man	Cytomegalovirus-induced gastrointestinal bleeding and pancreatitis	Type 2 diabetes mellitus, hypertension, atrial fibrillation, multivessel coronary artery disease, and primary cutaneous large B-cell lymphoma leg type	CPR: 263 mg/L, leukocytosis: 16,610/mm^3^, neutrophilia: 15,940/mm^3^,	Empirical treatment was started with hydroxychloroquine, lopinavir/ritonavir, methylprednisolone	NA	43	Cured
Kangas-Dick, Prien et al., 2020 [52]	74-year-old male	Gastrointestinal perforation	No	Serum albumin: 2.3 mg/dL WBCs: 15,900	Empirical treatment was started with hydroxychloroquine, azithromycin, and ceftriaxone	8–10 days	9 days	Dead
Farina, Rondi et al., 2020 [53]	70-year-old male	Acute bowel ischemia	No	WBCs: 15.3 × 10^3^ μ/L, CRP: 149 mg/L	No	3 days	2 days	Dead
Moazzam, Salim et al., 2020 [54]	4 months, 25-day-old baby boy	Intussusception	No	CRP: 3.7 mg/L, Ferritin: 162.9, D-Dimer: 3.8 mcg/L	Co-amoxiclav, amikacin, metronidazole, paracetamol	2 days	2.5 days	Cured
Paul Joy et al., 2021 [55]	66-year-old male	Ischemic colitis	No	Rapid drop in Hb to 4.7 g/dL	Azithromycin, cefuroxime, hydroxychloroquine, methylprednisolone, tocilizumab and convalescent plasma	3 days	40 days	Cured
Ibrahim, Karuppasamy et al., 2020 [56]	33-year-old man	Paralytic ileus	No	ALT: 1589 U/L, AST: 1856 U/L	Ceftriaxone, azithromycin, hydroxychloroquine, methylprednisolone, and a dose of tocilizumab	2 days	NA	In ICU
Ibrahim, Karuppasamy et al., 2020 [56]	33-year-old man	Paralytic ileus	No	Blood urea: 55.78 mmol/L, creatinine: 1891 μmol/L, bicarbonate: 1.9 mmol/L, lipase: >1200 U/L, amylase: 390 U/L, ALT: 187 U/L, AST: 125 U/L	Prokinetic agents and intravenous potassium replacement	NA	NA	Patient is currently on renal replacement therapy

Abbreviations: WBCs, white blood cells; CRP, C-reactive protein; LDH, lactate dehydrogenase; AST, aspartate aminotransferase; ALT, alanine aminotransferase; Hb, hemoglobin; TPC, total platelet count; TLC, total leukocyte count; NLR, neutrophil-to-lymphocyte ratio; PT, prothrombin time; INR, international normalized ratio; aPTT, activated partial thromboplastin clotting time; MCV, mean corpuscular volume; TIBC, total iron-binding capacity; GOT, aspartate aminotransferase blood test; GPT, alanine aminotransferase blood test.

### 4.2. Neurological System

#### 4.2.1. Complications

**Figure 2:**
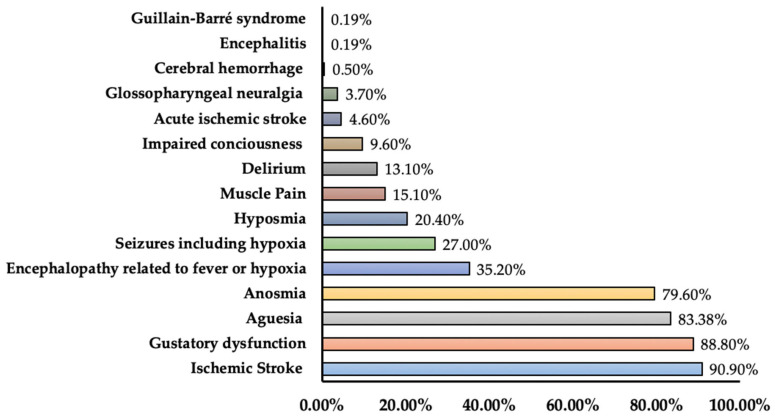
Prevalence of neurological complications among COVID-19 patients (prevalence represents maximum percentage reported in the literature).

Our search identified 14 research studies reporting neurological complications during the course of COVID-19. These studies reported 26 neurological complications. Major complications in research studies were acute cerebrovascular disease including acute ischemic stroke, cerebral venous sinus thrombosis, cerebral hemorrhage (IR: 0.5–90.9%), anosmia (IR: 4.9–79.6%), dysgeusia (IR: 2.8–83.38%), encephalopathy/encephalitis with or without fever and hypoxia (IR: 0.19–35.2%), and seizures (IR: 0.5–27%). Other complications noted were impaired consciousness (IR: 7.5–9.6%), myalgia/myopathy/muscle pain (incidence: 9.3%, 17.2%/3.1%/15.1%), ataxia/movement disorders (incidence: 0.5%/0.7%), ageusia (incidence: 1.7%, 2.8%) and hyposmia (incidence: 5.1%, 20.4%). Rare complications included a combination of dysgeusia and hyposmia (incidence: 3.4%), dysgeusia and anosmia (incidence: 3.4%), ageusia and hyposmia (incidence: 3.4%), ageusia and insomnia (incidence: 8.5%), gustatory dysfunction (incidence: 88.8%), electrolyte disturbances (hypokalemia: 13%, hyponatremia: 11%, hypocalcemia: 7%), delayed recovery of mental status after sedation (2.5%), delirium (13.1%), dysautonomia (2.5%), Guillain–Barré Syndrome (0.19%), optic neuritis (0.19%), trigeminal neuralgia (3.3%), glossopharyngeal neuralgia (3.7%), vasglossopharyngeal neuralgia (0.8%), Restless Leg Syndrome (1.7%), and nerve pain (2.3%). All-cause mortality in research studies reporting neurological complications ranged from 0.46% to 24.60%. One study specified 54.5% mortality among patients with cerebrovascular disease, and one study reported 4.1% mortality among patients with all neurological complications (Table 3). In Figure 2, the prevalence in terms of the maximum percentage of neurological complications among different studies is presented.

We also included 10 case reports comprising 11 patients indicating the involvement of neurological complications. These reports identified a total of nine neurological complications. Major complications seen in case reports were encephalopathy/encephalitis (27.27%), Guillian–Barré Syndrome (27.27%), and epileptogenicity/focal seizures with impaired awareness (18.18%). Other complications included focal temporal lobe dysfunction (9.09%), meningitis (9.09%), intracranial hemorrhage (9.09%), acute myelitis (9.09%), Miller Fisher Syndrome (9.09%), and polyneuritis cranialis (9.09%). Among the case reports, seven (63.64%) patients were cured, and two (18.18%) patients were still hospitalized and undergoing treatment. No patient among 11 of them died (Table 4).

#### 4.2.2. Underlying Pathophysiology

The underlying pathophysiological mechanisms proposed include direct invasion of neural parenchyma cells by the virus. The central nervous system is assessed by SARS-CoV-2 through the lamina cribrosa, olfactory bulb, and nasal mucosa or transport through the retrograde axonal. In the respiratory tree, ACE2 receptors are highly expressed in nasal epithelial cells [57,58], which may justify the symptoms of modified smell or taste mostly reported in outpatients retrospectively with SARS-CoV-2 [59,60,61]. The other complications are attributed to the neurovirulence of SARS-CoV-2, which reflects the prothrombotic and proinflammatory cascade resulting in cytokine storm [62], which in turn produces effects on the blood–brain barrier and brain vasculature. This has been particularly observed in critical patients experiencing toxic–metabolic prolongation of multi-organ dysfunction.

#### 4.2.3. Management Considerations

Specific management considerations for health practitioners include continuous conformity to the guidelines established for acute ischemic stroke (thrombectomy and thrombolysis) [63]. Guidelines for the monitoring of post-acute care for pandemic restrictions should be adopted. A remote video assessment, whenever feasible, should be considered for hospitalized patients with symptoms that might be related to stroke [64]. A delayed or extended-interval dosing of long-term immunomodulatory therapies should be considered in diseases such as multiple sclerosis in SARS-CoV-2 patients [65].

**Table 3:** Summary of research studies reporting neurological complications in COVID-19 patients.

Author Name, Year, Ref	Country	Type of Study	Demographic	Complication	Incidence	Mortality
Klopfenstein et al., 2020 [66]	France	Retrospective Observational	N = 54, Female: 36 (67%), Mean Age: 47 years (SD: 16)	Anosmia	47%	4% (Overall hospitalization rate 37%)
Bagheri et al., 2020 [67]	Iran	Retrospective Cross-sectional study	N = 10,069, Female: 7162 (71.13%), Mean Age: 32.5 years (Range: 7–78)	Anosmia	76.24%	NA
				Aguesia	83.38%	
Giacomelli et al., 2020 [68]	China	Retrospective Cross-sectional study	N = 69, Male: 40 (67.8%) Median Age: 60 years (SD: 15.9)	Dysgeusia	8.5%	NA
				Ageusia	1.7%	
				Hyposmia	5.1%	
				Dysgeusia and hyposmia	3.4%	
				Dysgeusia and anosmia	3.4%	
				Ageusia and hyposmia	3.4%	
				Ageusia and anosmia	8.5%	
Li et al., 2020 [69]	China	Retrospective Observational Cohort	N = 219, Female: 130 (59.4%) Mean Age: 53.3 (57–91)	Acute ischemic stroke	4.6%	NA
				Cerebral venous sinus thrombosis	0.5%	
				Cerebral hemorrhage	0.5%	
Lu et al., 2020 [70]	China	Retrospective Observational Cohort	N = 304, Male: 182 (59.9%), Mean Age: 44 years	Encephalopathy	2.65%	3.31%
				Seizures including hypoxia	27%	
				Electrolyte disturbances including		
				Hypokalemia	13%	
				Hyponatremia	11%	
				Hypocalcemia	7%	
Ling Mao, 2020 [71]	China	Retrospective Observational Case Series	N = 214, Male: 87 (40.7%), Mean Age: 52.7 years (SD: 15.5)	Impaired consciousness	7.5%	0.46%
				Acute cerebrovascular disease	2.8%	
				Ataxia	0.5%	
				Seizure	0.5%	
Corrado Lodigiani, 2020 [72]	Italy	Retrospective Cross-Sectional	N = 388, Male: 68%, Median Age (range): 66 years (55–75)	Acute ischaemic stroke	2.5%	23.71%
Yanan Li, 2020 [69]	China	Retrospective Observational	N = 21, Male: 62.5%, Age: Mean (SD) 52.7 ± 15.5	Ischemic stroke	90.9%	54.5% (with cerebrovascular disease)
				Intracranial hemorrhage	9.1%	
Jerome R. Lechien, 2020 [59]	Europe	Prospective Cohort	N = 417, Female: 263 (63.1%), Mean Age: 36.9 years (SD: 11.4)	Anosmia	79.6%	NA
				Hyposmia	20.4%	
				Gustatory dysfunction	88.8%	
Fernando Daniel Flores-Silva, 2021 [73]	Mexico	Prospective Cross-Sectional Observational	N = 221, Male: 59.3%, Age: Mean (SD); 36.9 ± 11.4	Delayed recovery of mental status after sedation	2.5%	24.6%
				Seizures	0.8%	
				Stroke	0.8%	
				Encephalitis	0.2%	
				Delirium	13.1%	
Carlos Manuel Romero-Sánchez, 2020 [74]	Spain	Retrospective Observational	N = 841, Male: 473 (56.2%), Mean Age: 66.42 years (SD: 14.96)	Myalgia	17.2%	4.1% (with neurologic complications)
				Anosmia	4.9%	
				Dysgeusia	6.2%	
				Disorders of consciousness	19.6%	
				Myopathy	3.1%	
				Dysautonomia	2.5%	
				Cerebrovascular disease	1.7%	
				Seizures	0.7%	
				Movement disorders	0.7%	
				Encephalitis	0.19%	
				Guillain–Barre syndrome	0.19%	
				Optic neuritis	0.19%	
Ömer Karadaş, 2020 [75]	Turkey	Prospective Cross-Sectional	N = 239, Male: 133 (55.6%), Mean Age: 46.46 years (SD: 15.41)	Impaired consciousness-confusion	9.60%	NA
				Muscle pain	15.1%	
				Cerebrovascular disorders	3.8%	
				Trigeminal neuralgia	3.3%	
				Glossopharyngeal neuralgia	3.7%	
				Vasoglossopharyngeal neuralgia	0.8%	
				Restless Leg Syndrome	1.7%	
Mao et al. 2020 [71]	China	Retrospective Observational Case Series	N = 214, Male: 87 (40.7%), Mean Age: 52.7 years (SD: 15.5)	Impaired consciousness	7.50%	NA
				Seizures	0.50%	
				Acute cerebrovascular disease	2.80%	
				Anosmia	5.10%	
				Dysgeusia	5.60%	
				Nerve pain	2.3%	
Marco Luigetti, 2020 [76]	Italy	Retrospective Observational	N = 213, Male: 137 (64.32%), Mean Age: 70.2 (SD: 13.9)	Encephalopathy related to fever or hypoxia	35.2%	18.7%
				Encephalopathy not related to fever or hypoxia	5.1%	
				Ageusia/dysgeusia	2.8%	
				Anosmia/hyposmia	6.1%	
				Seizure	2.8%	
				Ischemic stroke	0.9%	
				Hemorrhagic stroke	0.9%	
				Encephalitis	0.5%	
				Myalgia	9.3%	

**Table 4:** Summary of case studies reporting neurological complications in COVID-19 patients.

Name of Author, Year, Ref	Demographics	Type of Complication	Past History	Laboratory Tests	Treatment	Days of Illness before Hospitalization	Hospital Stays	Outcome
Filatov et al., 2020 [17]	74-year-old male	Encephalopathy, focal temporal lobe dysfunction, and epileptogenicity	Chronic obstructive pulmonary disease (COPD), Parkinson’s disease, cardioembolic stroke, atrial fibrillation, cellulitis	Chest X-ray showed small right pleural effusion as well as bilateral ground-glass opacities. CT of chest revealed patchy bibasilar consolidations and subpleural opacities. CT scan of head showed area of encephalomalacia in the left temporal region	Antiepileptic medication, vancomycin, meropenem, acyclovir, hydroxychloroquine and lopinavir/ritonavir	NA	NA	Patient was still in the ICU, Critically ill with poor prognosis
Moriguchi et al., 2020 [77]		Meningitis/encephalitis	No	Blood investigation demonstrated high levels of WBCs, neutrophil dominance, and increased CRP. The CSF cell count was 12/μL	Intravenous (IV) ceftriaxone, steroids, aciclovir, and vancomycin. Intravenous administration of Levetiracetam, Favipiravir was also administered	NA	NA	Treatment continued
Poyiadji et al., 2020 [78]	58-year-old female	Acute hemorrhagic necrotizing encephalopathy	No	CT scan of head showed bilateral medial thalami. Brain MRI showed hemorrhagic rim	Intravenous immunoglobulin	3 days	NA	NA
Gutiérrez-Ortiz et al., 2020 [79]	50-year-old male	Miller Fisher syndrome	Bronchial asthma	Neuro-ophthalmological testing showed right internuclear ophthalmoparesis along with right fascicular oculomotor palsy. Lymphopenia: 1000 cells/μL, CRP: 2.8 mg/dL, CSF examination opening pressure of 11 cm H_2_O	IV immunoglobulin 0.4 g/kg for 5 days	2 days	2 weeks	Ataxia and cranial neuropathies improved, Discharged
Gutiérrez-Ortiz et al., 2020 [79]	39-year-old male	Polyneuritis cranialis	No	Neuro-ophthalmological exam showed fixed nystagmus. severe abduction deficits were also observed in both eyes	Treated symptomatically with acetaminophen	3 days	NA	Discharged, Complete recovery afterward
Sharifi-Razavi et al., 2020 [80]	79-year-old male	Intracranial hemorrhage	No	Lymphopenia: 590 cells/mm^2^, ESR: 85 mm/h, CRP 10 mg/L, creatinine: 1.4 mg/dL, platelets: 210 × 10^9^/L, PT: 12 s, INR: 1, PPT: 64 s	NA	3 days	NA	NA
Paybast et al., 2020 [81]	38-year-old male	Guillain–Barré Syndrome	Hypertension, upper respiratory tract infection	Nerve conduction study was performed, which demonstrated considerable decrease in the amplitude of compound motor action potentials	Therapeutic plasma exchange, labetalol by intravenous bolus along with hydroxychloroquine sulfate orally	5 days	NA	Discharged
Bozzali et al., 2021 [82]	54-year-old female	Focal seizures with impaired awareness	No	Brain MRI scan which showed white matter hyperintensities on T2-weighted images. CSF examination showed a slight elevation of proteins (55 mg/dL) while cell count was normal	Levetiracetam therapy showed partial benefit so treatment was shifted to carbamazepine	NA	NA	Cured
H. Zhao et al., 2020 [83]	61-year-old female	Guillain–Barré Syndrome	No	Lymphocytopenia: 0·52 × 10^9^/L, thrombocytopenia: 113 × 10^9^/L	Intravenous immunoglobulin, rbidol, lopinavir, and ritonavir	1 day	30 days	Cured and discharged
K. Zhao et al., 2020 [84]	66-year-old male	Acute myelitis	No	Decreased RBCs, Hb, serum total protein, serum albumin, elevated CRP, ALT, AST, and CK.	Ganciclovir, lopinavir/ritonavir, moxifloxacin, meropenem, glutathione, dexamethasone, human immunoglobulin, mecobalamin, and pantoprazole	2 days	NA	Cured and discharged
Sedaghat and Karimi 2020 [85]	65 years- old male	Guillain–Barré syndrome	Type 2 diabetes mellitus	Serum glucose: 159 mg/dL, BUN: 19 mg/dL, creatinine: 0.8 mg/dL, ALT: 35 IU/L, AST: 47 IU/L, WBC count: 14,700 cells per microliter; ESR: 72 mm/hour, CRP: 2+, Hb: 11.6 g/dL	Hydroxychloroquine, lopinavir/ritonavir (LPV/RTV), and azithromycin	5 days	9 days	Cured

Abbreviations: WBCs, white blood cells; RBCs, red blood cells; CRP, C-reactive protein; Hb, hemoglobin; BUN, bloodurea nitrogen; CT, computed tomography; AST, aspartate aminotransferase; ALT, alanine aminotransferase; CK, creatine kinase; ESR, erythrocyte sedimentation rate; MRI, magnetic resonance imaging; CSF, cerebrospinal fluid; PT, prothrombin time; PPT, partial thromboplastin time.

### 4.3. Renal System

#### 4.3.1. Complications

**Figure 3:**
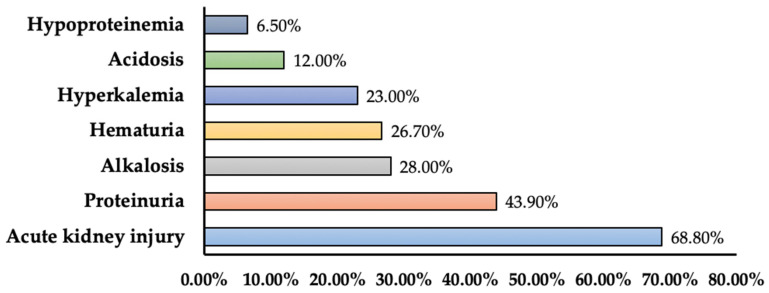
Prevalence of renal complications among COVID-19 patients (prevalence represents maximum percentage reported in the literature).

We included 16 studies reporting renal complications among COVID-19 patients. A total of seven renal complications were noted in research studies. The major complication was acute kidney injury (AKI)/acute renal failure (IR: 0.5–68.8%). Other complications included electrolyte disturbances (incidence: 7.2%, 23%), acidosis (incidence: 9%, 12%), proteinuria (incidence: 6.5%, 43.9%), hematuria (incidence: 26.7%), alkalosis (incidence: 28%) and continuous renal replacement therapy clotting due to hypertriglyceridemia. All-cause mortality ranged from 1.4% to 52.4%, whereas 9% to 93.6% of patients were still hospitalized due to at least one complication. Mortality rates caused by AKI/kidney disease ranged between 16.1% and 66.35%, and patients still hospitalized due to them varied significantly. In one retrospective cohort study, 50% of non-survivors died due to acute kidney injury [86]. In one retrospective case series, mortality was observed among patients with acidosis (12%), alkalosis (28%), AKI (25%), and hyperkalemia (23%) [87] (Table 5). In Figure 3, the prevalence in terms of the maximum percentage of renal complications among different studies was presented.

We included seven case reports comprising seven patients in the current review. These case reports reported a total of eight renal complications. The major complications identified were renal/splenic/cerebral infarct or aortic thrombosis (57.14%). Rare complications were catastrophic thrombotic syndrome (14.29%), glomerulonephritis (14.29%), polycystic kidney disease (14.29%), IgA neuropathy (14.29%), and spinal epidural abscess (14.29%). Of the seven patients in these case reports, 71.43% (n = 5/7) were cured/improved, one patient died, and one patient was discharged but not completely cured (Table 6).

#### 4.3.2. Underlying Pathophysiology

Several possible pathophysiological mechanisms have been identified for renal abnormalities. Firstly, the virus may infect the renal cells directly, as evidenced by the existence of ACE2 receptors on them and histopathological findings [88,89,90]. Secondly, microvascular dysfunction is incidental to endothelial damage as demonstrated by renal lymphocytic endothelialitis, and moreover to the inclusion of particles of the virus in the endothelium of glomerular capillary cells [30]. Thirdly, cytokine storm may have a vital role in the immunopathology of AKI [91]. Another likely mechanism for glomerular injury is arbitrated by specific immunological effector mechanisms induced by the virus or viral antigen immunocomplexes [88]. In addition, proteinuria is not considered the classic manifestation of AKI; short-term heavy albuminuria might arise from direct podocyte injury or endothelial dysfunction. Other causative etiologies for renal injury include acute respiratory distress syndrome, interstitial nephritis, volume depletion, and rhabdomyolysis [92].

#### 4.3.3. Management Considerations

Specific management considerations by health practitioners for managing renal complications should include evaluation of the albumin-to-creatinine ratio and complete urine analysis, providing the coalition of hematuria and proteinuria with the outcomes [93,94]. Empirical systemic low-dose anticoagulants, when initiating and also in the routine management of RRT extracorporeal circuits, should be considered [95]. Consideration should be taken to co-localize patients requiring RRT, and a shared protocol for RRT should be used [96]. Additionally, acute peritoneal dialysis among select patients should be considered so as to minimize the requirement of personnel [96].

**Table 5:** Summary of research studies reporting renal complications in COVID-19 patients.

Author Name, Year, Ref	Country	Type	Demographic	Complication	Incidence	Mortality
Hirsch, Ng et al., 2020 [97]	USA	Observational Cohort Study	N = 5449, Male: 3317 (60.87%) Median Age: 64.0 years (IQR: 52–75)	AKI	36.6%	35% (Among AKI patients) 39% patients still admitted to hospital.
Guan, Ni et al., 2020 [98]	China	Observational Cohort Study	N = 1099, Male: 637 (57.96%) Median Age: 56 years (IQR: 46–67)	AKI	0.5%	1.4% 93.6% patients still admitted to hospital
Yan, Zuo et al., 2021 [99]	China	Observational Cohort Study	N = 882, Male: 440 (49.89%) Median Age: 71 years (IQR: 68–77)	AKI	13%	59.1% (Among AKI cohort) 24.3% AKI patients were still admitted to hospital
Cheng, Luo et al., 2020 [93]	China	Prospective Cohort Study	N = 701, Male: 367 (52.35%) Median Age: 63 years (IQR: 50–71)	AKI Proteinuria Hematuria	5.1% 43.9% 26.7%	16.1% (with kidney disease)
Abramovitz et al., 2023 [100]	USA	Retrospective Case Series	N = 11	Continuous renal replacement therapy clotting due to hypertriglyceridemia	73% related to propofol use causing hypertriglyceridemia 27% due to total parenteral nutrition administration	NA
Aggarwal et al., 2020 [101,102]	USA	Retrospective Cross-Sectional	N = 16, Male: 12 (75%), Median Age: 67 years (Range: 38–95)	AKI	68.8%	19%
Arentz et al., 2020 [102]	USA	Retrospective Cross-Sectional	N = 21, Male: 52%, Mean Age: 70 years (Range: 43–92)	Acute kidney failure	19.1%	52.4%
Tao Guo et al., 2020 [103]	China	Retrospective Case Series	N = 187, Male: 91 (48.7%) Mean Age: 58.50 years (SD: 14.66)	AKI	14.6%	23%
Chaolin Huang et al., 2020 [104]	China	Prospective Cross-Sectional	N = 41, Male: 30 (73%), Median Age: 49·0 years (IQR: 41·0–58·0)	AKI	7%	15% 17% patients still admitted to hospital
Richardson et al., 2020 [105]	USA	Retrospective Cross-Sectional	N = 5700, Male: 3437 (60.3%), Median Age: 63 years (IQR: 52–75)	AKI	22.2%	66.35% (Among AKI patients), 27.63% AKI patients still admitted to hospital
Chan et al., 2021 [106]	USA	Retrospective Observational	N = 3993, Female:1704 (43%), Median Age: 64 years (IQR: 56–78)	AKI	46%	50% (Among AKI patients)
Mohamed et al., 2020 [107]	USA	Prospective Observational	N = 575, Female: 263 (45.74%), Median Age: 65 years (IQR: 36–96)	AKI	28%	50% (Among AKI cohort)
Zhou et al., 2020 [86]	China	Retrospective Cohort	N = 191, Male: 119 (62%), Median Age: 56 years (IQR: 46–67)	AKI Acidosis	15% 9%	50% (Among non-survivors)
Argenziano et al., 2020 [108]	USA	Retrospective Case Series	N = 1000, Male: 596 (59.6%), Median Age: 63.0 (IQR: 50.0–75.0)	AKI	34%	9% 21.1% still admitted to hospital
Chen et al., 2020 [87]	China	Retrospective Case Series	N = 274, Male: 171 (62%), Median Age; 62.0 years (IQR: 44.0–70.0)	Acidosis	12%	14% (Among acidosis patients) 40% (Among alkalosis patients) 25% (Among AKI patients 37% (Among hyperkalemia patients)
				Alkalosis	28%	
				AKI	11%	
				Hyperkalemia	23%	
Shi et al., 2020 [109]	China	Retrospective Cohort	N = 416, Female: 211 (50.7%), Median Age: 64.0 years (Range: 21–95)	Electrolyte disturbance	7.2%	13.7%, 76.1% still admitted to hospital
				AKI	1.9%	
				Hypoproteinemia	6.5%	

Abbreviation: AKI, acute kidney injury.

**Table 6:** Summary of case studies reporting renal complications in COVID-19 patients.

Name of Author, Year, Ref	Country	Age, Gender	Type of Complication	Past History	Laboratory Tests	Treatment	Days of Illness before Hospitalization	Hospital Stays	Outcome
Moeinzadeh, Dezfouli et al., 2020 [110]	Iran	25 years, Male	Glomerulonephritis	No	Creatine: 4.2, CRP: 2+, ESR: 120 mm/h, Hb: 4.5 g/dL, WBC: 4500/mm^3^, protein in urine analysis = 3+	1 g of methylprednisolone IV, plasmapheresis with PLASMART, Versatile™ PES, 3 doses intravenous immunoglobulin (IVIG), 20 g each time	2 days	17 days	Cured
Haque, Jahan et al., 2020 [111]	Bangladesh	68 years, Male	Polycystic kidney disease	Nephrolithotomy	Hb: 9.2 gm/dL, thrombocytopenia: 112,000/cm^2^, serum creatinine: 3.3 mg/dL, urea: 90 mg/dL	Anticoagulation along with linagliptin, aspirin, atorvastatin	N/R	More than 30 days	Expired
Mavraganis et al., 2022 [112]	Greece	64 years, Male	Renal infarct, splenic infarct, aortic thrombosis	No	Lymphopenia: 950 lymphocytes), ALT: 130 U/L, AST: 135 U/L, D-dimers:0.57 mg/L, hs-CRP: 19.9 mg/dL, CK: 1215 U/L, LDH: 551 U/L	Low-molecular-weight heparin at prophylactic dose, intravenous dexamethasone and remdesivir, omeprazole, tocilizumab, and ceftaroline	2 weeks	19 days	Discharged, Not cured completely
Göre et al., 2022 [113]	Turkey	56 years, Male	IgA neuropathy and spinal epidural abscess	No	Creatinine: 3.64 mg/dL, GFR: 18 mL/min/1.73 m^2^, leukocytes: 9.9 × 10^−9^/L, lymphocytes: 1.87× 10^−9^/L, neutrophils: 7.12 × 10^−9^/L, platelets: 382 × 10^−9^/L, Hb: 6.9 g/dL	Teicoplanin and ciprofloxacin, methylprednisolone at dose of 1 mg/kg/day	Almost one month	25 days	Marked improvement in complications
Rigual et al., 2022 [114]	Spain	53 years, Male	Cerebral, splenic, and renal infarction	No	D-dimer: 850 ng/dL, IL-6: 8.60 pg/dL, ferritin: 499 ng/dL fibrinogen: 541 mg/dL, CRP: 7.8 mg/dL,	LMWH 1 mg subcutaneous enoxaparin/kg/24 h along with ASA methylprednisolone 1 mg/kg/24 h	16 days	30 days	Cured
Brem et al., 2022 [115]	Morocco	59 years, Male	Renal and splenic infarct with catastrophic thrombotic syndrome	Diabetes mellitus	WBCs: 15,000 elements/mm^3^, lymphopenia: 450 elements/mm^3^, platelets: 120,000 elements/mm^3^, D-dimer level: 33,620 ng/mL, fibrinogen: 4.5 g/L, CRP: 189.88 mg/L, ferritin: 4150 ng/mL, LDH: 1221 unit/L, CK level: 20,500 U/L, creatinine level: 9.59 mg/L	LMWH 60 mg twice a day after embolectomy. Infracondylar amputation of the right lower limb afterward	NA	NA	Cured
Gjonbalaj et al., 2020 [116]	Kosovo	50 years, Male	Renal artery thrombosis	No	CRP: 4.3 mg/dL, troponin: 2.3 pg/mL, urea: 29.96 g/dL, creatinine: 1.2 mg/dL, D-dimer: 142 ng/mL, CK-MB: 2.2 ng/mL.	Thrombus aspiration plus bolus administration of tirofiban. Oral anticoagulant therapy	2 days	3 days	Cured

Abbreviations: CRP, C-reactive protein; LDH, lactate dehydrogenase; Hb, hemoglobin; ALT, alanine aminotransferase; AST, aspartate aminotransferase; hs-CRP, high-sensitivity C-reactive protein; GFR, glomerular filtration rate; CK, creatine kinase; CK-MB, creatine kinase–myoglobin binding; LMWH, low-molecular-weight heparin; ASA, acetyl salicylic acid.

### 4.4. Cardiovascular System

#### 4.4.1. Complications

**Figure 4:**
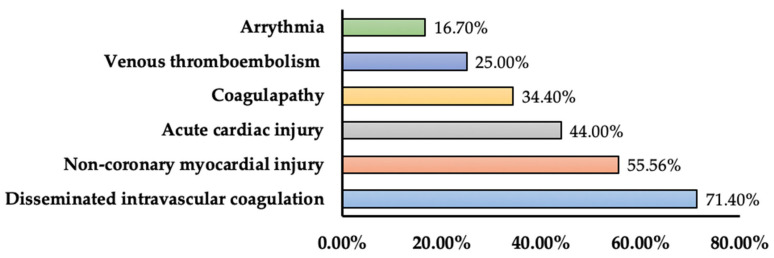
Prevalence of cardiovascular complications among COVID-19 patients (prevalence represents maximum percentage reported in the literature).

We have included 18 research studies reporting cardiovascular complications. In total, 15 complications were uncovered from these research studies. The major complications in research studies included acute cardiac injury/non-coronary myocardial injury (IR: 7.2–55.56%), arrhythmia/ventricular tachycardia/ventricular fibrillation (IR: 5.9–16.7%), coagulopathy/venous thromboembolism (IR: 19–34.4%), and myocardial infarction/heart failure (IR: 23.0–44.44%). Other complications included acute coronary syndrome and cardiac insufficiency (incidence: 0.96% and 17.4%). In one study, disseminated intravascular coagulation was seen in 71.4% of the non-survivors. All-cause mortality ranged from 4.3% to 45%, and among in-hospital patients, it ranged from 6.7% to 76.7%. In one retrospective case series, 77% of acute cardiac injury patients and 49% of heart failure patients did not survive [87] (Table 7). In Figure 4, the prevalence in terms of the maximum percentage of cardiovascular complications among different studies was presented.

We have also included 11 case reports (11 patients) reporting cardiovascular complications. From case reports, a total of 10 cardiovascular complications were identified. Major complications in case reports were fulminant myocarditis (27.27%) and Takotsubo syndrome (18.18%). Other complications included myopericarditis complicated by cardiac tamponade (9.09%) and isolated hemorrhagic pericardial effusion with tamponade (9.09%). Of the twelve patients in case reports, five patients (33.33%) improved/were cured and were discharged from the hospitals, while two patients (16.67%) died (Table 8).

#### 4.4.2. Underlying Pathophysiology

The underlying pathophysiology of CV complications is most likely multifactorial. ACE2 is highly expressed in endothelial cells, fibroblasts, and myocytes of the CV tissue [90,117], and direct viral injury is one possible mechanism. Myocarditis, MI, and circulatory failure may develop due to viral load and inflammatory infiltrates, as evidenced by some autopsy studies [118,119,120] and pathological reports [121,122]. In addition, endothelial damage mediated by viruses [30] and cytokine storms can be another assumed underlying mechanism for myocardial injury [123]. In general, viral infections further predispose patients to MI [124], and this risk is elevated in COVID-19 patients, with evidence of unjustifiably escalated hypercoagulability, which induces MI mediated by thrombotic events. Furthermore, isolated right ventricular malfunction may arise due to pulmonary thromboembolism [125,126] and increased pulmonary vascular pressures due to ARDS [127].

#### 4.4.3. Management Considerations

Important management considerations for CV complications by health practitioners should not include the discontinuation of angiotensin-converting enzyme inhibitors or angiotensin-receptor blockers in patients who are already using them at home, and assessment should be made on the basis of individual patient condition [128,129]. Furthermore, those who have torsades de pointes risk and are treated with drugs for QTc prolongation should be monitored by telemetry, and an electrocardiogram should be performed [130]. Above all, in order to minimize the viral transmission risk, the utility of diagnostic modalities (endomyocardial biopsies, invasive hemodynamic assessments, and cardiac imaging) should be considered carefully [131,132]. The preferred approach for most patients with ST-segment elevation MI is primary percutaneous coronary intervention. Furthermore, fibrinolytic therapy in specific patients should be considered if personal protective supplies are unavailable [133,134,135].

**Table 7:** Summary of research studies reporting cardiovascular complications in COVID-19 patients.

Author Name, Year, Ref	Country	Type of Study	Demographic	Complication	Incidence	Mortality
Huang et.al., 2020 [104]	China	Prospective Cross-Sectional	N = 41, Male: 30 (73%) Median Age: 49 years (IQR: 41.0–58.0)	Acute cardiac injury	12%	15% 17% still admitted to hospital
Dawei Wang et al., 2020 [136]	China	Retrospective Case Series	N = 138, Male: 75 (54.3%) Median Age: 56 years (IQR: 42–68)	Arrhythmia Acute cardiac injury	16.7% 7.2%	4.3%
Hong et al., 2020 [137]	South Korea	Retrospective Cross-Sectional	N = 98, Female: 60 (61.2%) Mean Age: 55.4 years (SD: 17.1)	Acute cardiac injury	11.2%	5.1% 58.2% still admitted to hospital
Tao Guo et al., 2020 [103]	China	Retrospective Case Series	N = 187, Male: 91 (48.7%) Mean Age: 58.50 years (SD: 14.66)	Ventricular tachycardia/ventricular fibrillation Coagulopathy	5.9% 34.4%	23%
Shaobo Shi et al., 2020 [109]	China	Retrospective Cohort	N = 416, Female: 211 (50.7%) Mean Age: 64 years (Range: 21–95)	Myocardial injury	19.7%	13.7% 76.7% still admitted to hospital
Zhou et al., 2020 [86]	China	Retrospective Cohort	N = 191, Male: 119 (62%) Median Age: 56.0 years (IQR: 46.0–67.0)	Heart failure Coagulopathy Acute cardiac injury	23% 19% 17%	28.27%
Songping Cui et al., 2020 [138]	China	Retrospective Cross-Sectional	N = 81, Female: 44 (54%) Mean Age: 59.9 years (Range 32–91),	Venous thromboembolism	25%	9.87% (All were VTE patients) Overall 11% patients remained in hospital
Chen et al., 2020 [87]	China	Retrospective Case Series	N = 274, Male: 171 (62%), Median Age: 62.0 years (IQR: 44.0–70.0)	Acute cardiac injury Heart failure	44% 24%	77% (Among acute cardiac injury patients) 49% (Among heart failure patients)
Yu Y et al., 2020 [139]	China	Prospective Cross-Sectional	N = 226, Male 139 (61.5%) Age: 64 (IQR: 57–70)	Cardiac injury Arrhythmia	27% 9.3%	38.5% 6.7% of overall patients were still admitted to hospital
Victoria L. Cammann et al., 2020 [140]	Italy, Spain, and Switzerland	Retrospective Cohort	N = 45, Male: 37 (82.2%), Mean Age: 69.7 years (SD: 11.1)	Acute coronary syndromes	0.96%	27.3% (Among COVID-19-positive acute coronary syndrome patients)
Zhou et al., 2020 [141]	China	Retrospective Cross-Sectional	N = 254, Male: 115 (45.28%) Mean Age: 50.6 years (Range: 15–87)	Acute heart failure Arrthymias	2.4% 6.3%	6.3%
Tang et al., 2020 [142]	China	Retrospective Cohort	N = 183, Male 98 (54%) Mean Age: 54.1 years (Range: 14–94)	Disseminated intravascular coagulation	71.4% (Among non-survivors)	11.5%
Ruan et al., 2020 [143]	China	Retrospective Cohort	N = 150, Female 48 (32%) Mean Age: Died = 67 years (15–81), Discharged = 50 years (44–81)	Among death group: Myocardial damage (some with fulminant myocarditis) Respiratory failure + myocardial damage (some with fulminant myocarditis)	7% 33%	45%
Lang Wang et al., 2020 [144]	China	Retrospective Cross-Sectional	N = 339, Female: 173 (51.0%), Median Age: 69 years (Range: 65–76)	Acute cardiac injury Arrhythmia Cardiac insufficiency	21% 10.4% 17.4%	19.2%
K. Liu et al., 2020 [145]	China	Retrospective Cross-Sectional	N = 137, Female: 76 (55.47%) Median Age: 57 years (Range: 20–83 years)	Arrhythmia	7.3%	11.7% (Among patients with complications)
Wang et al., 2020 [136]	China	Retrospective Case Series	N = 138, Male: 75 (54.3%) Age Median:56 years (IQR: 42–68)	Arrhythmia Acute cardiac injury	16.7% 7.2%	4.3%
Wei JF et al., 2020 [146]	China	Prospective Cohort	N = 101, Male: 54 (53.5%) Age: 49 (IQR: 34–62)	Acute myocardial injury	15.8%	2.97% (Among acute myocardial injury)
Sripal Bangalore et al., 2020 [147]	USA	Retrospective Case Series	N = 18, Male: 15 (83%) Median Age: 63 years (Range: 54–73)	ST-segment elevation Myocardial infarction Non-coronary myocardial injury	100% 44.44% 55.56%	50% (Among myocardial infarction patients) 90% (Among non-coronary myocardial injury patients)

**Table 8:** Summary of case studies reporting cardiovascular complications in COVID-19 patients.

Name of Author, Year, Ref	Age, Gender	Type of Complication	Past History	Laboratory Tests	Treatment	Days of Illness before Hospitalization	Hospital Stays	Outcome
Richard, Robinson et al., 2020 [148]	28-year-old female	Fulminant myocarditis	Diabetes mellitus type 1, diabetic gastroparesis, asthma, anxiety, depression	WBCs: 29 × 10^3^/μL, Hb: 10.3 g/dL, hematocrit: 31%, creatinine: 4.4 mg/dL, glucose: 1679 mg/dL, potassium: 2.9 mmol/L, lactic acid level: 17.1 mg/dL, CRP: 2.47 mg/dL, LDH: 296 U/L, ferritin: 119 ng/mL, troponin: 0.04 ng/mL	IV methylprednisolone 1 g daily for three days, IV steroids	NA	NA	Patient clinically improved
Zeng et al., 2020 [149]	63-year-old male	Fulminant myocarditis Disseminated intravascular coagulation	Allergic cough Smoking	NT-proBNP 750 pg/mL, IL-6: 7.63 pg/mL. troponin I: 0.10 g/L, Candida, human α-herpesvirus, and β-herpesvirus were also detected	Lopinavir–ritonavir, methylprednisolone, interferon α-1b, piperacillin–tazobactam immunoglobulin	NA	33 days	Died
Fiore et al., 2021 [150]	45-year-old male	Cardiogenic shock Myocarditis	No	Hs-troponin T: normal, lactate: normal, NT-proBNP: 945 pg/dL, CRP: normal, left ventricular ejection fraction: 40–45%	Hydroxychloroquine 200 mg bid, broad-spectrum empirical antibiotic therapy	4 days	Almost a month	Discharged
Hu, Ma et al., 2021 [151]	37-year-old male	Myocarditis	Chest pain, dyspnea, diarrhea	Increased BNP and CK-MB, ECG: ST↑ (III and AVF), no coronary stenosis in CT angiography	Methylprednisolone (200 mg/day, 4 days), immunoglobulin (20 g/day, 4 days), norepinephrine, diuretic (toracemide and furosemide), milrinone, piperacillin sulbactam, pantoprazole	3 days	NA	NA
Hua et al., 2020 [152]	47-year-old female	Myopericarditis complicated by cardiac tamponade	No	Troponin T levels were 225 and 253 ng/L	IV fluid resuscitation, vasopressor support	NA	NA	NA
Dabbagh et al., 2020 [153]	67-year-old female	Isolated hemorrhagic pericardial effusion with tamponade	Nonischemic cardiomyopathy with left ventricular ejection fraction	Hs-troponin I: <18 ng/L, BNP: 54 pg/mL CRP: 15.9 mg/dL, ferritin: 593 ng/mL, D-dimer: 6.52 mg/mL, interleukin-6: 8 pg/mL	Hydroxychloroquine along with colchicine and glucocorticoid	NA	NA	Discharged
Danzi et al., 2020 [154]	75-year-old female	Acute pulmonary embolism	No	Leucocytosis: 11.360/mm^2^, CRP: 180 mg/L, troponin I: 3240.4 ng/mL, D-dimer: 21 μg/mL	LMWH, lopinavir/ritonavir, and hydroxychloroquine	10 days	NA	NA
Meyer et al., 2020 [155]	83-year-old female	Takotsubo syndrome	Chronic hypertension	Cardiac troponin T: 1142 ng/L	Conventional heart failure medication	3 days	10 days	Discharged
Minhas et al., 2020 [156]	58-year-old women	Takotsubo syndrome	Diabetes mellitus type 2, hypertension, and dyslipidemia	Troponin I level: 11.02 ng/mL, lymphocyte count: 1.04 K/mm^3^	Hydroxychloroquine, dobutamine	5 days	NA	Not cured completely but discharged
Jud et al., 2021 [157]	24-year-old women	Vascular reactivity and Arterial stiffness	No	Flow-mediated dilation: 0.0%, nitroglycerin-mediated dilation: 17.24%, aortic pulse-wave analysis: 5.6 m/s, ultrasonography revealed augmentation index: 13%, and carotid intima–media thickness of 0.4 mm	NA	NA	NA	NA
Xu et al., 2020 [122]	50-year-old male	Cardiac arrest	No	WBCs: 6.28 × 10^9^/L, Hb: 134.00 g/L, latelet count: 205.00 × 10^9^/L, Creatininse: 67 μmol/L Pressure of oxygen in arterial blood: 28 mmHg, PT: 14.9 s	Interferon alfa-2b, lopinavir plus ritonavir as antiviral therapy, and moxifloxacin, methylprednisolone (80 mg twice daily, intravenously)	8 days	6 days	Died

Abbreviations: BNP, B-type natriuretic peptide; CRP, C-reactive protein; LDH, lactate dehydrogenase; Hb, hemoglobin; WBCs, white blood cells; NT-proBNP, N-terminal prohormone of brain natriuretic peptide; CK-MB, creatine kinase–myoglobin binding; PT, prothrombin time; hs-troponin T, high-sensitivity troponin T; ECG, electrocardiogram; ST, sinus tachycardia; AVF, arteriovenous fistula.

## 5. Limitations and Strengths

There are several limitations in this review that must be taken into account. First, we did not perform a meta-analysis due to the large heterogeneity and variations in data collection and study designs. Secondly, a quality assessment of the studies was not performed. Thirdly, it might be possible that a confirmed diagnosis of these complications is lacking in primary studies. Lastly, due to the large body of available literature, complications related to only four major organ systems were covered in the current review. There are certain strengths of the present review that cannot be neglected. Firstly, according to our knowledge, this is the first review that points out the unusual complications in key organ systems after COVID-19. Secondly, the compilation of a large amount of evidence in a single article provides information on the range of incidences of atypical complications caused by the COVID-19 virus and their impact on hospitalization and mortality rates. Thirdly, the findings of the current review highlight the importance of the consideration of other organ systems during the management of COVID-19 infection.

## 6. Future Prospects

Since the commencement of the SARS-CoV-2 pandemic, an exceeding number of evidence has concentrated on the quick diagnosis, evolution, and divergence of new therapies. Nevertheless, it has been found in various studies, including the current review, that SARS-CoV-2 is not just a respiratory disease. Elevated levels of endogenous chemical substances generated in reaction to inflammation developed by the virus have the potential to generate disturbances and alterations in target tissues all over the human body, which even surpass the protective barriers of innate tissue immunity. Moreover, the cytokine storm developed during sepsis, which also has pleiotropic capabilities, interacts with respective high-density receptors, vasculature, and immune cells. In addition, the overexpression of angiotensin-converting enzyme Ⅱ receptors (hACE2-R) in numerous tissues permits the virus to proliferate to the vascular system and extend into the entire human body. A vicious cycle develops, which entails the generation of chemical mediators, a decrease in the density of the hACE2-R receptors, and elevated levels of angiotensin Ⅱ, producing both inflammatory and vascular effects. Moreover, this mechanism prompts the generation of additional hACE2-R via positive feedback. These frequently stimulated cycles multiply the proliferation of infection and consequent expansion in angiotensin Ⅱ, which widely contribute to the pathophysiological mechanisms of SARS-CoV-2 and generate increased inflammation, vasoconstriction, and fibrosis. This necessitates the focus of healthcare practitioners on not only the respiratory syndrome caused by this virus but also on monitoring for atypical complications in all major body systems and following up on patients for post-COVID and long-COVID health issues, which at times are entirely asymptomatic in nature. Hence, there is a need for a multidisciplinary approach. Moreover, further research is required to identify actual differences as there is a wide variability across studies. Also, the impact of novel variants on long-COVID development and which individuals will be at the most risk will need future study and research.

## 7. Conclusions

This article reviewed four major organ systems to determine the burden of atypical complications. Major gastric complications found in research studies are bowel ischemia/infarction, GI bleeding, and hepatic ischemia/injury/infarct due to thromboembolism of the portal system. Major neurological complications included acute ischemic stroke, cerebral venous sinus thrombosis, cerebral hemorrhage, anosmia, and dysgeusia. Major renal complications included acute kidney injury (AKI) and acute renal failure. Major cardiovascular complications included acute cardiac injury/non-coronary myocardial injury, arrhythmia/ventricular tachycardia/ventricular fibrillation, coagulopathy/venous thromboembolism, and myocardial infarction/heart failure. The major complications found in these organ systems in case reports were bowel ischemia/hepatic ischemia, acute pancreatitis, bowel perforation, encephalopathy/encephalitis, Guillain–Barré Syndrome, epileptogenicity/focal seizures with impaired awareness, renal/splenic/cerebral infarct or aortic thrombosis, fulminant myocarditis, and Takotsubo syndrome. Hence, it is important for healthcare practitioners, researchers, and policy-makers to opt for early management practices and long-term follow-up of COVID-19 patients, consider evidence-based research practices, and compel effective policy-making, which would help drive the global community toward the successful management of the after-effects of the COVID-19 pandemic.

## Data Availability

Not applicable.
